# Supplementary material for: Heat adaptation of phage T7 under an extended genetic code
Source: Virus Evol. 2021 Dec 1;7(2):veab100. doi: 10.1093/ve/veab100 (PMC8923235; doi:10.1093/ve/veab100)
Supplement: veab100_Supp [file veab100_supp.zip › Sequencing_Analysis.zip!/Sequencing_Analysis/A1/evidence/JC_SIDE_1_53.html]

BRESEQ :: Evidence


| New junction evidence | | | | | | | | | | | |
| --- | --- | --- | --- | --- | --- | --- | --- | --- | --- | --- | --- |
|  | | seq id | position | reads (cov) | reads (cov) | score | skew | freq | annotation | gene | product |
| \* | ? | NC\_001604.1 | = 9789 | 3293 (1.200) | 306 (0.110) +CGACGAAGAAAGCTGGGACGAAGA | 4/486 | 42.4 | 8.3% | intergenic (–/–) | *–/–* | –/– |
|  | ? | NC\_001604.1 | 9790 = | 3613 (1.170) |  |  |  |  | intergenic (–/–) | *–/–* | –/– |
| Rejected: Coverage evenness skew score above cutoff. | | | | | | | | | | | |
| Rejected: Frequency below/above cutoff threshold. | | | | | | | | | | | |

Only 100 of 3920 total aligned reads displayed.  

`GCGTCTTTCCAAGACAAGAAGACCAAAGAGACCAAGCACATCAATCTGGTTGTGGTTGACTCAAAAGGTAAGAAGATGGAAGACGTTCCGATTATCGGTGGTGGCTCTAAGCTGAAAGTTAAATATTCTCTGGTTCCATACAAGTGGAACACTGCTGTAGGTGCGAGCGTTAAGCTGCAACTGGAATCCGTGATGCTGGTCGAACTGGCTACCTTTGGTGGCGGTGAAGACGATTGGGCTGACGAAGTTGAAGAGAACGGCTATGTTGCCTCTGGTTCTGCCAAAGCGAGCAAACCACGCGACGAAGAAAGCTGGGACGAAGACGACGAAGAGTCCGAGGAAGCAGACGAAGACGGAGACTTCTAAGTGGAACTGCGGGAGAAAATCCTTGAGCGAATCAAGGTGACTTCCTCTGGGTGTTGGGAGTGGCAGGGCGCTACGAACAATAAAGGGTACGGGCAGGTGTGGTGCAGCAATACCGGAAAGGTTGTCTACTGTCATCGCGTAATGTCTAATGCTCCGAAAGGTTCTACCGTCCTGCACTCCTGTGATAATCCATTATGTTGTAACCCTGAACACCTATCCATAGGAACTCCAAA  >  NC_001604.1/9491‑10089`  

                                                                                                                                                                                                                                                                                                          `|`                                                                                                                                                                                                                                                                                                              
           `agacaagaAGACCAAAGAGACCAAGCACATCAATCTGGTTGTGGTTGACTCAAAAGGTAAGAAGATGGAAGACGTTCCGATTATCGGTGGTGGCTCTAAGCTGAAAGTTAAATATTCTCTGGTTCCATACAAGTGGAACACTGCTGTAGGTGCGAGCGTTAAGCTGCAACTGGAATCCGTGATGCTGGTCGAACTGGCTACCTTTGGTGGCGGTGAAGACGATTGGGCTGACGAAGTTGAAGAGAACGGATATGTTGCCTCTGGTTCTGCCAAAGCGAGCAAACCACGCGACGAAGAAAGc                                                                                                                                                                                                                                                                                                 <  1:453847/301‑1 (MQ=255)`  
                `agaagaCCAAAGAGACCAAGCACATCAATCTGGTTGTGGTTGACTCAAAAGGTAAGAAGATGGAAGACGTTCCGATTATCGGTGGTGGCTCTAAGCTGAAAGTTAAATATTCTCTGGTTCCATACAAGTGGAACACTGCTGTAGGTGCGAGCGTTAAGCTGCAACTGGAATCCGTGATGCTGGTCGAACTGGCTACCTTTGGTGGCGGTGAAGACGATTGGGCTGACGAAGTTGAAGAGAACGGCTATGTTGCCTCTGGTTCTGCCAAAGCGAGCAAACCACGCGACGAAGAAAGCTGGGa                                                                                                                                                                                                                                                                                            <  1:119237/301‑1 (MQ=255)`  
                `agaagaCCAAAGAGACCAAGCACATCAATCTGGGTGTGGTTGACTCAAAAGGTAAGAAGATGGAAGACGTTCCGATTATCGGTGGTGGCTCTAAGCTGAAAGTTAAATATTCTCTGGTTCCATACAAGTGGAACACTGCTGTAGGTGCGAGCGTTAAGCTGCAACTGGAATCCGTGATGCTGGTCGAACTGGCTACCTTTGGTGGCGGTGAAGACGATTGGGCTGACGAAGTTGAAGAGAACGGCTATGTTGCCTCTGGTTCTGCCAAAGCGAGCAAACCACGCGACGAAGAAAGCTGGGa                                                                                                                                                                                                                                                                                            <  1:26578/301‑1 (MQ=255)`  
                           `gagaCCAAGCACATCAATCTGGTTGTGGTTGACTCAAAAGGTAAGAAGATGGAAGACGTTCCGATTATCGGTGGTGGCTCTAAGCTGAAAGTTAAATATTCTCTGGTTCCATACAAGTGGAACACTGCTGTAGGTGCGAGCGTTAAGCTGCAACTGGAATCCGTGATGCTGGTCGAACTGGCTACCTTTGGTGGCGGTGAAGACGATTGGGCTGACGAAGTTGAAGAGAACGGCTATGTTGCCTCTGGTTCTGCCAAAGCGAGAAAACCACGCGACGAAGAAAGCTGGGACGAAGacgacg                                                                                                                                                                                                                                                                                 >  1:53316/1‑301 (MQ=255)`  
                            `agaCCAAGCACATCAATCTGGTTGTGGTTGACTCAAAAGGTAAGAAGATGGAAGACGTTCCGATTATCGGTGGTGGCTCTAAGCTGAAAGTTAAATATTCTCTGGTTCCATACAAGTGGAACACTGCTGTAGGTGCGAGCGTTAAGCTGCAACTGGAATCCGTGATGCTGGTCGAACTGGCTACCTTTGGTGGCGGTGAAGACGATTTGGCTGACGAAGTTGAAGAGAACGGCTATGTTGCCTATGGTTCTGCCAACTCGAGCAAACCACGCTACGACGAAAGCTGGGACGAAGAcgacga                                                                                                                                                                                                                                                                                >  1:261915/1‑301 (MQ=255)`  
                                      `caTCAATCTGGTTGTGGTTGACTCAAAAGGTAAGAAGATGGAAGACGTTCCGATTATCGGTGGTGGCTCTAAGCTGAAAGTTAAATATTCTCTGGTTCCATACAAGTGGAACACTGCTGTAGGTGCGAGCGTTAAGCTGCAACTGGAATCCGTGATGCTGGTCGAACTGGCTACCTTTGGTGGCGGTGAAGACGATTGGGCTGACGAAGTTGAAGAGAACGGCTATGGTGCCTCTGGTTCTGCCAAAGCGAGCAAACCACGCGACGAAGAAAGCTGGGACGAAGACGACGAAGAGTCCGAg                                                                                                                                                                                                                                                                      >  1:449830/1‑301 (MQ=255)`  
                                           `aTCTGGTTGTGGTTGACTCAAAAGGTAAGAAGATGGAAGACGTTCCGATTATCGGTGGTGGCTCTAAGCTGAAAGTTAAATATTCTCTGGTTCCATACAAGTGGAACACTGCTGTAGGTGCGAGCGTTAGGCTGCAACTGGAATCCGTGATGCTGGTCGAACTGGCTACCTTTGGTGGCGGTGAAGACGATTGGGCTGACGAAGTTGAAGAGAACGGCTATGTTGCCTCTGGTTCTGCCAAAGCGAGAAAACCACGCGACGAAGAAAGCTGGGACGAAGACGAAGAAGAGTCCGAGGAAGc                                                                                                                                                                                                                                                                 >  1:28365/1‑301 (MQ=255)`  
                                           `aTCTGGTTGTGGTTGACTCAAAAGGTAAGAAGATGGAAGACGTTCCGATTATCGGTGGTGGCTCTAAGCTGAAAGTTAAATATTCTCTGGTTCCATACAAGTGGAACACTGCTGTAGGTGCGAGCGTTAAGCTGCAACTGGAATCCGTGATGCTGGTCGAACTGGCTACCTTTGGTGGCGGTGAAGACGATTGGGCTGACGAAGTTGAAGAGAACGGACATGTTGCCTCTGGTTCTGCCAAAGCGAGCAAACCACGAGACGAAGAAAGCTGGGACGAAGACGACGAAGAGTCCGAGGAAgg                                                                                                                                                                                                                                                                 >  1:367791/1‑300 (MQ=255)`  
                                               `ggttgtggttgACTCAAAAGGTAAGAAGATGGAAGACGTTCCGATTATCGGTGGTGGCTCTAAGCTGAAAGTTAAATATTCTCTGGTTCCATACAAGTGGAACACTGCTGTAGGTGCGAGCGTTAAGCTGCAACTGGAATCCGTGATGCTGGTCGAACTGGCTACCTTTGGTGGCGGTGAAGACGATTGGGCTGACGAAGTTGAAGAGAACGGCTATGTTGCCTCTGGTTCTGCCAAAGCGAGCAAACCACGCGACGAAGAAAGCTGGGACGAAGACGACGAAGAGTCCGAGGAAGCAGAc                                                                                                                                                                                                                                                             >  1:57716/1‑301 (MQ=255)`  
                                               `ggctcggagatgtgtataagagacagagaTGGAAGACGTTCCGATTATCGGTGGTGGCTCTAAGCTGAAAGTTAAATATTCTATGGTTCCATACAAGTGGAACACTGCTGTAGGTGCGAGCGTTAAGCTGCAACTGGAATCCGTGATGCTGGTCGAACTGGCTACCTTTGGTGGCGGTGAAGACGATTGGGCTGACGAAGTTGAAGAGAACGGCTATGTTGCCTCTGGTTCTGCCAAAGCGAGCAAACCACGCGACGAAGAAAGCTGGGACGAAGACGACGAAGAGTCCAAGGAAGCAGAc                                                                                                                                                                                                                                                             <  1:380779/275‑1 (MQ=255)`  
                                                   `ggggttgACTCAAAAGGGAAGAAGATGGAGGACTGTCCGATTATCGGTGGTGGGTCTAAGCTGAAAGTTAAAGATTCTCTGGTTCCATACAAGTGGAACACTGCTGTAGGTGCGAGCGTTAAGCTGCAACTGGAATCCGTGATGCTGGTCGAACTGGCTACCTTTGGTGGCGGTGAAGACGATTGGGCTGACGAAGTTGAAGAGAACGGCTATGTTGCCTCTGGTTCTGCCAAAGCGAGCAAACCACGCGACGAAGAAAGCTGGGACGAAGACGACGAAGAGTCCGAGGAAGCAGACGAAg                                                                                                                                                                                                                                                         <  1:245708/299‑1 (MQ=255)`  
                                                           `atCAAAATGTAAGAAGATGGAAGACGTTCCGATTATCGGTGGTGGCTCTAAGCTGAAAGTTAAATATTCTCTGGTTCCATACAAGTGGAACACTGCTGTAGGTGCGAGCTTTAAGCTGTAACTGGAATCCGTGATGCTGGTCGAACTAGCTACCTTTGGTGGCGGTGAAGACGATTGGGCTGACGAAGTTGAAGAGAACGGCTATGTTGCCTCTGGTTCTGCAAAAGCGAGCAAACCACGCGACGAAGAAAGCTGGGACGAAGACGACGAAGAGTCCGAGGAAGCAGACGAAGACGGAGAc                                                                                                                                                                                                                                                 <  1:190532/300‑1 (MQ=255)`  
                                                                  `ggTAATAAGATGGAAGACGTTCCGATTATCGGTGGTGGATCTAAGCTGAAAGTTAAATATTCTCTGGTTCCATACAAGTGGAACACTGCTGTAGGTGCGAGCGTTAAGCTGCAACTGGAATCCGTGATGCTGGTCGAACTGGCTACCTTTGGTGGCGGTGAAGACGATTGGGCTGACGAAGTTGAAGAGAACGGCTATGTTGCCTCTGGTTCTGCCAAAGCGAGCAAACCACGCGACGAAGAAAGCTGGGACGAAGACGACGAAGAGTCCGAGGAAGCAGACGAAGACGGAGACTTCTAAg                                                                                                                                                                                                                                          >  1:90833/1‑301 (MQ=255)`  
                                                                  `ggTAAGAAGATGGAAGATGTTCCGATTATCGGTGGTGGCTCTAAGCTGAAAGTTAAATATTCTCTGGTTCCATACAAGTGGAACACTGCTGTAGGTGCGAGCGTTAAGCTGCAACTGGAATCCGTGATGCTGGTCGAACTGGCTACCTTTGGTGGCGGTGAAGACGATTGGGCTGACGAAGTTGAAGAGAACGGCTATGTTGCCTCTGGTTCTGCCAAAGCGAGCAAACCACGCGACGAAGAAAGCTGGGACGAAGACGACGAAGAGTCCGAGGAAGCAGACGAAGACGGAGACTTCTAAg                                                                                                                                                                                                                                          >  1:249747/1‑301 (MQ=255)`  
                                                                  `ggTAAGAAGATGGAAGACGTTCCTATTATCGGTGGTGGCTCTAAGCTGAAAGTTAAATATTCTCTGGTTCCATACAAGTGGAACACTGCTGTAGGTGCGAGCGTTAAGCTGCAACTGGAATCCGTGATGCTGGTCGAACTGGCTACCTTTGGTGGCGGTGAAGACGATTGGGCTGACGAAGTTGAAGAGAACGGCTATGTTGCCTCTGGTTCTGACAAAGCGAGCAAACCACGCGACGAAGAAAGCTGGGACGAAGACGACGCAGAGTCCGAGGAAGCAGACGAAGACGGAGACTTCTAAg                                                                                                                                                                                                                                          >  1:440444/1‑301 (MQ=255)`  
                                                                   `gTAAGAAGATGGAAGACGTTCCGATTATCGGGGGTGGCTCTAAGCTGAAAGTTAAATATTCTCTGGTTCCATACAAGTGGAACACTGCTGTAGGTGCGAGCGTTAAGCTGCAACTGGAATCCGTGATGCTGGTCGAACTGGCTACCTTTGGTGGCGGTGAAGACTATTGGGCTGACGAAGTTGAAGAGAACGGCTATGTTGCCTCTGGTTCTGCCAAAGCGAGCAAACCACGCGACGAAGAAAGCTGGGACGAAGACGACGAAGAGTCCGAGGAAGCAGACGAAGACGGAGACTTCTAAGt                                                                                                                                                                                                                                         <  1:147947/301‑1 (MQ=255)`  
                                                                   `gTAAGAAGATGGAAGACGTTCCGATTAGCGGTGGTGGCTCTAAGCTGAAAGTTAAATATGCTCTGGTTCCATACAAGTGGAACACTGCTGTAGGTGCGAGCGTTAAGCTGCAACTGGAATCCGTGATGCTGGTCGAACTGGCTACCTTTGGTGGCTGTGAAGACGATTGGGCTGACGAAGTTGAAGAGAACGGCTATGTTGCCTCTGGTTCTGCCAAAGCGAGCAAACCACGCGACGAAGAAAGCTGGGACGAAGACGACGAAGAGTCCGAGGAAGCAGACGAAGACGGAGACTTCTAAGt                                                                                                                                                                                                                                         <  1:200361/301‑1 (MQ=255)`  
                                                                     `aagaagATGGAAGACGTTCCGATTATCGGTGGTGGCTCTAAGCTGAAAGTTAAATATTCTCTGGTTCCATACAAGTGGAACACTGCTGTAGGTGCGAGCGTTAAGCTGCAACTGGAATCCGTGATGCTGGTCGAACTGGCTACCTTTGGTGGCGGTGAAGACGATTGGGCTGACGAAGTTGAAGAGAACGGCTATGTTGCCTCTGGTTCTGCCAAAGCGAGCAAACCACGCGACGAAGAAAGCTGGGACGAAGACGACGAAGAGTCCGAGGAAGCAGACGAAGACGGAGACTTCTAAGTgg                                                                                                                                                                                                                                       <  1:423202/301‑1 (MQ=255)`  
                                                                         `agaTGGAAGACGTTCCGATTATCGGTGGTGGCTCTAAGCTGAAAGTTAAATATTCTCTGGTTCCATACAAGTGGAACACTGCTGTAGGTGCGAGCGTTAAGCTGCAACTGGAATCCGTGATGCTGGTCGAACTGGCTACCTTTGGTGGCGGTGAAGACGATTGGGATGACGAAGTTGAAGAGAACGGCCATGTTGCCTCTGGTTCTGCCAAAGCGTGCAAACCACTCGGCGAAGAAAGCTGGGACGAAGACGACGAAGAGTCCGAGGAAACAGACGAAGACGGAGACTTCTAAGTGGaagt                                                                                                                                                                                                                                   >  1:86773/1‑299 (MQ=255)`  
                                                                          `taTGGAAGACGTTCCGATTATCGGTGGTGGCTCTAAGCTGAAAGTTAAATATTCTCTGGTTCCATACAAGTGGAACACTGCTGTAGGTGCGAGCGTTAAGCTGCAACTGGAATCCGTGATGCTGGTCGAACTGGCTACCTTTGGTGGCGGTGAAGACGATTGGGCTGACGAAGTTGAAGAGAACGGCTATGTTGCCTCTGGTTCTGCCAAAGCGAGCAAACCACGCGACGAAGAAAGCTGGGACGAAGACGACGAAGAGGCCGAGGAAGCAGACGAAGACGGAGACTTCTAAGTGGAACTg                                                                                                                                                                                                                                  >  1:115540/2‑301 (MQ=255)`  
                                                                          `gaTGGAAGACGTTCCGATTATCGGTGGTGGCTCTAAGCTGAAAGTTAAATATTCTCTGGTTCCATACAAGTGGAACACTGCTGTAGGTGCGAGCGTTAAGCTGCAACTGGAATCCGTGATGCTGGTCTAACTGGCTACCTTTGGTGGCGGTGAAGACGCGTGGGCTGACGAAGGTGAAGAGAACGGCTATGTTGCATCTGGTTCTGCCACAGCGAGCAAACGACGCGACGAAAAAAGCTGGGACGAAGACGAGGAAGAGTCCGAGGAAGCAGAAGAAGAAGGAGACTTCTAAGTGGAAcag                                                                                                                                                                                                                                  >  1:351812/1‑299 (MQ=255)`  
                                                                          `gaTGGAAGACGTTCCGATTATCGGTGGTGGCTCTAAGCTGAAAGTTAAATATTCTCTGGTTCCATACAAGTGGAACACTGCTGTAGGTGCGAGCGTTAAGCTGCAACTGGAATCCGTGATGCTGGTCGAACTGGCTACCTTTGGTGGCGGTGAAGACGATTGGGCTGACGAAGTTGAAGAGAACGGCTATGTTGCCTCTGGTTCTGCCAAAGCGAGCAAACCACGCGACGAAGAAAGCTGGGACGAAGACGACGAAGAGTCCGAGGAAGCAGACGAAGACGGAGACTTCTAAGtctgtctc                                                                                                                                                                                                                                  >  1:220105/1‑294 (MQ=255)`  
                                                                                   `cGTTCCGATTATCGGTGGTGGCTCTAAGCTGAAAGTTAAATATTCTCTGGTTCCATACAAGTGGAACACTGCTGTAGGTGCGAGCGTTAAGCTGCAACTGGAATCCGTGATGCTGGTCGAACTGGCTACCTTTGGTGGCGGTGAAGACGATTGGGCTGACGAAGTTGAAGAGAACGGCTATGTTGCCTCTGGTTCTGCCAAAGCGAGCAAACCACGCGACGAAGAAAGCTGGGACGAAGACGACGAAGAGTCCGAGGCAGCAGACGAAGACGGAGACTTCAAAGTGGAACTGCGGGAGaaa                                                                                                                                                                                                                         >  1:215675/1‑301 (MQ=255)`  
                                                                                    `gTTCCGATTATCGGTGGTGGCTCTAAGCTGAAAGTTAAATATTCTCTGGTTCCATACAAGTGGAACACTGCTGTAGGTGCGAGCGTTAAGCTGCAACTGGAATCCGTGATGCTGGTCGAACTGGCTACCTTTGGTGGCGGTGAAGACGATTGGGCTGACGAAGTTGAAGAGAACGGCTATGTTGCCTCTGGTTCTGCCAAAGCGAGCAAACCACGCGACGAAGAAAGCTGGGACGAAGACGACGAAGAGTCCGAGGAAGCAGACGAAGACGGAGACTTCTAAGTGGAACTGCGGGAGaaaa                                                                                                                                                                                                                        >  1:275743/1‑301 (MQ=255)`  
                                                                                     `agagacatagtggtggtGGCTATAAACGGAAAGTTAAATATTCTCGTGTTCCATACAAGTGGAACACTGCTGTAGGGGCGAGCGTTAAGCTGAAACTGGAATCCGGGATGCTGGTCGAACTGGCTACCTTTGGTGGCGGTGAAGACGATTGGGCTGACGAAGTTGAAGAGAACGGCTATGTTGCCTCTGGTTCTGCCAAAGCTAGCAAACCACGCGACGAAGAAAGCTGGGACGAAGACGACGAAGAGTCCGATGAAGCAGACGAAGACGGAGACTTCTAAGTGGAACTGCGGGAGAAAAt                                                                                                                                                                                                                       <  1:67961/290‑1 (MQ=255)`  
                                                                                         `gATTATCGGTGGTGGCTCTAAGCTGAAAGTTAATTATTCTCTGGTTCCATACAAGTGGAACACTGCTGTAGGTGCGAGCGTTAAGCTGCAACTGGAATCCGTGATGCTGGTCGAACTGGCTACCTTTGGTGGCGGTGAAGACGATTGGGCTGACGAAGTTGAAGAGAACGGCTATGTTGCCTCTGGTTCTTCCAAAGCGAGCAAACCACGCGACGAAGAAAGCTGGGACGAAGACGACGAAGAGTCCGAGGAAGCAGACGAAGACTGAGACTTCTAAGTGGAACTGCggctgtctcttata                                                                                                                                                                                                                   >  1:103698/1‑289 (MQ=255)`  
                                                                                             `aTCGGTGGTGGCTCTAAGCTGAAAGTTAAATATTCTCTGGTTCCATACAAGTGGAACACTGCTGTAGGTGCGAGCGTTAAGCTGCAACTGGAATCCGTGATGCTGGTCGAACTGGCTACCTTTGGTGGCGGTGAAGACGATTGGGCTGACGAAGTTGAAGAGAACGGCTATGTTGCCTCTGGTTCTGCCAAAGCGAGCAAACCACGCGACGAAGAAAGCTGGGACGAAGACGACGAAGAGTCCGAGGAAGCAGACGAAGACGGAGACTTCTAAGTGGAACTGCGGGAGAAAATCCTTGAga                                                                                                                                                                                                               >  1:347982/1‑300 (MQ=255)`  
                                                                                                      `ggCTCTAAGCTGAAAGTTAAATATTCTCTGGTTCCATACAAGTGGAACACTGCTGTAGGTGCGAGCGTTAAGCTGCAACTGGAATCCGTGATGCTGGTCGAACTGGCTACCTTTGGTGGCGGTGAAGACGATTGGGCTGACGAAGTTGAAGAGAACGGCTATGTTGCCTCTGGTTCTGCCAAAGCGAGCAAACCACGCGACGAAGAAAGCTGGGACGAAGACGACGAAGAGTCCGAGGAAGCAGACGAAGACGGAGACTTCTAAGTGGAACTGCGGGAGAAAATCCTTGAGCGAATCAAgg                                                                                                                                                                                                      <  1:322881/301‑1 (MQ=255)`  
                                                                                                       `gCTCTAATCTGAAAGTTAAATATTCTCTGGTTCCATACAAGTGGAACACTGCTGTAGGTGCGAGCGTTAAGCTGCAACTGGAATCCGTGATGCTGGTCGAACTGGCTACCTTTGGTGGCGGTGAAGACGATTGGGCTGACGAAGTTGAAGAGAACGGCTATGTTGCCTCTGGTTCTGCCAAAGCGAGCAAACCACGCGACGAAGAAAGCTGGGACGAAGACGACGAAGAGTCCGAGGAAGCAGACGAAGACGGAGACTTCTAAGTGGAACTGCGGGAGAAAATCCTTGAGCGAATCAAGGt                                                                                                                                                                                                     >  1:271491/1‑301 (MQ=255)`  
                                                                                                                     `ggacagtatTCTCTGGTTCCATACAAGTGGAACACTGCTGTAGGTGCGAGCGTTAAGCTGCAACTGGAATCCGTGATGCTGGTCGAACTGGCTACCGTTGGTGGCGGTGAAGACGATTGGGCTGACGAAGTTGAAGAGAACGGCTATGTTGCCTCTGGTTCTGCCAAAGCGAGCAAACCACGCGACGAAGAAAGCTGGGACGAAGACGACGAAGAGTCCGAGGAAGCAGACGAAGACGGAGACTTCTAAGTGGAACTGCGGGAGAAAATCCTTGAGCGAATCAAGGTGACTTCCTATGGgt                                                                                                                                                                                       <  1:469650/295‑1 (MQ=255)`  
                                                                                                                          `atatTCTCTGGTTCCATACAAGTGGAACACTGCTGTAGGTGCGAGCGTTAAGCTGCAACTGGAATCCGTGATGCTGGTCGAACTGGCTACCTTTGGTGGCGGTGAAGACGATTGGGCTGACGAAGTTGAAGAGAACGGCTATGTTGCCTCTGGTTCTGCCAAAGCGAGCAAACCACGCGACGAAGAAAGCTGGGACTAAGACGACGAAGAGTCCGAGGAAGCAGACGAAGACGGAGACTTCTAAGTGGAACTGCGGGAGAAAATCCTTGAGCGAATCAAGGTGACTTCctcctgtctctta                                                                                                                                                                                  >  1:332809/1‑291 (MQ=255)`  
                                                                                                                           `aatTATCGGGTTCCAGAAAAGTGGAACAATGCTGTAAGAGCGAGCAGTAAGCTGAAACTGGAATCGGTGATGCTGGTCGAACTGGCTACCTGTGGTGGCGGTGAAGACGATTGTGCTGACGAAGTTGAAGAGAACGGCGATGTTGCCTCTGGATCTGCCAAAGCGAGCAAACCACGCGACGAAGAAAGCTGTGACGAAGACGACGAAGAGTCAGAGGAAGCAGACGCAGACGGAGACTGCTAAGTGGAACTGCGGGAGAAAATCCTTGAGCGAATCAAGGTGACTTCCTCTGGGTGTTggg                                                                                                                                                                                 <  1:445450/300‑1 (MQ=255)`  
                                                                                                                            `atTCTCTGGTTCCATACAAGTGGAACACTGCTGTAGGTGCGAGCGTTAAGCTGCAACTGGAATCCGTGATGCTGGTCGAACTGGCTACCTTTGGTGGCGGTGAAGACGATTGGGATGACGAAGTTGAAGAGAACGGCTATGTTGCCTCTGGTTCTGCCAAAGCGAGCAAACCACGCGACGAAGAAAGCTGGGACGAAGACGACGAAGAGTCCGAGGAAGCATACGAAGACGGAGACTTCTAAGTGGAACTGCGGGAGAAAATCCTTGAGCGAATCAAGGTGACTTCCTCTGGGTGCTgtct                                                                                                                                                                                >  1:314562/1‑298 (MQ=255)`  
                                                                                                                             `ttCTCTGGTTCCATACAAGTGGAACACTGCTGTAGGTGCGAGCGTTAAGCTGCAACTGGAATCTGTGATGCTGGTCGAACTGGCTACCTTTGGTGGCGGTGAAGACGATTGGGCTGACGAAGTTGAAGAGAACGGCTATGTTGCCTCTGGTTCTGCAAAAGCGAGCAAACCACGCGACGAAGAAAGCTGGGACGAAGACGACGAAGAGTCCGAGGAAGCAGACGAAGACGGAGACTTCTAAGTGGAACTGCGGGAGAAAATCCTTGAGCGAATCAAGGTGACTTCCTCTGGGTGTTGGGAg                                                                                                                                                                               <  1:337177/301‑1 (MQ=255)`  
                                                                                                                               `ctctGGTTCCATACAAGTGGAACACTGCTGTAGGTGCGAGCGTTAAGCTGCAACTGGAATCCGTGATGCTGGTCGAACTGGCTACCTTTGGTGGCGGTGAAGACGATTGGGCTGACGAAGTTGAAGAGAACGGCTATGTTGCCTCTGGTTCTGCCAAAGCGAGCAAACCACGCGACGACGAAAGCTGGGACGAAGACGACGAAGAGTCCGAGGAAGCAGACGAAGACGGAGACTTCTAAGTGGAACTGCGGGAGACAATCCTTGAGCGAATCAAGGTGACTTCCTCTGGGTGTTGGGAGTg                                                                                                                                                                             >  1:158391/1‑301 (MQ=255)`  
                                                                                                                               `ctctGGTTCCATACAAGTGGAACACTGCTGTAGGTGCGAGCGTTAAGCTGCAACTGGAATCCGTGATGCTGGTCGAACTGGCTACCTTTGGTGGCGGTGAAGACGATTGGGCTGACGAAGTTGAAGAGAACGGCTATGTTGCCTCTGGTTCTGCCAAAGCGAGCAAACCACGCGACGAAGAAAGCTGGGACGAAGACGACGAAGAGTCCGAGGAAGCAGACGAAGACGGAGACTTCTAAGTGGAACTGCGGGAGAAAATCCTTGAGCGAATCAAGGTGACTTCCTCTGGGTGTTGGGAGTg                                                                                                                                                                             >  1:418033/1‑301 (MQ=255)`  
                                                                                                                               `ctctGGTTCCATACAAGTGGAACACTGCTGTAGGTGCGAGCGTTAAGCTGCAACTGGAATCCGTGATGCTGGTCGAACTGGCTACCTTTGGTGGCGGTGAAGACGATTGGGCTGACGAAGTTGAAGAGAACGGCTATGTTGCCTCTGGTTCTGCCAAAGCGAGCAAACCACGCGACGAAGAAAGCTGGGACGAAGACGACGAAGAGTCCGAGGAAGCAGACGAAGACGGAGACTTCTAAGTGGAACTGCGGGAGAAAATCCTTGAGCGAATCAAGGTGAATTCCTCTGGGGGTTGGGAgcg                                                                                                                                                                             >  1:210344/1‑299 (MQ=255)`  
                                                                                                                                   `ggTTCCATACAAGTGGAACACTGCTGTAGGTGCGAGCGTTAAGCTGCAACTGGAATCCGTGATGCTGGTCGAACTGGCTACCTTTGGTGGCGGTGAAGACGATTGGGCTGACGACGTTGAAGAGAACGGCTATGTTGCCTCTGGTTCTGCCAAAGCGAGCAAACCACGCGACGAAGAAAGCTGGGACGAAGACGACGAAGAGTCCGAGGAAGAAGACGAAGACGGAGACTTCTAAGTGGAACTGCGGGAGAAAATCCTTGAGCGAATCAAGATGACTTCCTCTGGGTGTTGGGAGTGGCAg                                                                                                                                                                         >  1:487861/1‑301 (MQ=255)`  
                                                                                                                                    `gTTCCATACAAGTGGAACACTGCTGTAGGTGCGAGCGTTAAGCTGCAACTGGAATCCGTGATGCTGGTCGAACTGGCTACCTTTGGTGGCGGTGAAGACGATTGGGCTGACGAAGTTGAAGAGAACGGCTATGTTGCCTCTGGTTCTGCCAAAGCGAGCAAACCACGCGACGAAGAAAGCTGGGACGAAGACGACGAAGAGTCCGAGGAAGCAGACGAAGACGGAGACTTCTAAGTGGAACTGCGGGAGAAAATCCTTGAGCGAATCAAGGTGACTTCCTCTGGGTGTTGGGAGTGGCggg                                                                                                                                                                        >  1:317533/1‑301 (MQ=255)`  
                                                                                                                                    `gTTCCATACAAGTGGAACACTGCTGTAGGTGCGAGCGTTAAGCTGCAACTGGAATCCGTGATGCTGGTCGAACTGGCTACCTTTGGTGGCGGTGAAGACGATTGGGCTGACGAAGTTGAAGAGAACGGCTATGTTGCCTCTGGTTCTGCCAAAGCGAGCAAACCACGCGACGAAGAAAGCTGGGACGAAGACGACGAAGAGTCCGAGGAAGCAGACAAAGACGGAGACTTCTAAGTGGAACTGCGGGAGAAAATCCTTGAGCGAATCAAGGTGACTTCCTCTGGGTGTTGGGAGTGGCAgg                                                                                                                                                                        >  1:465007/1‑301 (MQ=255)`  
                                                                                                                                       `ccATACAAGTGGAACACTGCTGTAGGTGCGAGCGTTAAGCTGCAACTGGAATCCGTGATGCTGGTCGAACTGGCTACCTTTGGTGGCGGTGAAGACGATTGGGCTGACGAAGTTGAAGAGAACGGCTATGTTGCCTCTGGTTCTGCCAAAGCGAGCAAACCACGCGACGAAGAAAGCTGGGACGAAGACGACGAAGAGTCCGAGGAAGCAGACGAAGACGGAGACTTCTAAGTGGAACTGCGGGAGAAAATCCTTGAGCGAATCAAGGTGACTTCCTCTGGGTGTTGGGAGtctgtctctt                                                                                                                                                                     >  1:279844/1‑292 (MQ=255)`  
                                                                                                                                         `aTACAAGTGGAACACTGCTGTAGGTGCGAGCGTTAAGCTGCAACTGGAATCCGTGATGCTGGTCGAACTGGCTACCTTTGGTGGCGGTGAAGACGATTGGGCTGACGAAGTTGAAGAGAACGGCTATGTTGCCTCTGGTTCTGCCAAAGCGAGCAAACCACGCGACGAAGAAAGCTGGGACGAAGACGACGAAGAGTCCGAGGAAGCAGACGAAGACGGAGACTTCTAAGTGGAACTGCGGGAGAAAATCCTTGGGCGAATCAAGGTGACTTCCTCTGGGTGTTGGGAGTGGCAGGGCGCt                                                                                                                                                                   >  1:428608/1‑301 (MQ=255)`  
                                                                                                                                               `gTGGAACACTGCTGTAGGTGCGAGCGTTAAGCTGCAACTGGAATCCGTGATGCTGGTCGAACTGGCTACCTTTGGTGGCGGTGAAGACGATTGGGCTGACGAAGTTGAAGAGAACGGCTATGTTGCCTCTGGTTCTGCCAAAGCGAGCAAACCACGCGACGAAGAAAGCTGGGACGAAGACGACGAAGAGTCCGAGGAAGCAGACGAAGACGGAGACTTCTAAGTGGAACTGCGGGAGAAAATCCTTGAGCGAATCAAGGTGACTTCGTCTGGGTGTTGGGAGTGGCAGGgctgtctctta                                                                                                                                                             >  1:81587/1‑292 (MQ=255)`  
                                                                                                                                               `gTGGAACACTGCTGTAGGTGCGAGCGTTAAGCTGCAACTGGAATCCGTGATGCTGGTCGAACTGGCTACCTTTGGTGGCGGTGAAGACGATTGGGCTGACGAAGTTGAAGAGAACGGCTATGTTGCCTCTGGTTCTGCCAAAGCGAGCAAACCACGCGACGAAGAAAGCTGGGACGAAGACGACGAAGAGTCCGAGGAAGCAGACGAAGACGGAGACTTCTAAGTGGAACTGCGGGAGAAAATCCTTGAGCGAATCAAGGTGACTTCCTCTGGGTGTTGGGAGTGGCAGGGCGCTACGAAc                                                                                                                                                             >  1:176257/1‑301 (MQ=255)`  
                                                                                                                                                  `gAACACTGCTGTAGGTGCGAGCGTTAAGCTGCAACTGGAATCCGTGATGCTGGTCGAACTGGCTACCTTTGGTGGCGGTGAAGACGATTGGGCTGACGAAGTTGAAGAGAACGGCTATGTTGCCTCTGGTTCTGCCAAAGCGAGCAAACCACGCGACGAAGAAAGCTGGGACGAAGACGACGAAGAGTCCGAGGAAGCAGACGAAGACGAAGACTTCTAAGTGGAACTGCGGGAGAAAATCCTTGAGCGAATAAAGGTGACTTCCTCTGGGTGTTGGGAGTGGCAGGGCGCTACGAACAAt                                                                                                                                                          >  1:100053/1‑301 (MQ=255)`  
                                                                                                                                                    `gacaggctgTAGGTGCGAGCGTTAAGCTGCAACTGGAATCCGTGATGCTGGTCGAACTGGCTACCTTTGGTGGCGGTGAAGACAATTGGGCTGACGAAGTTGAAGAGAACGGCTATGTTGCCTCTGGTTCTGCCAAAGCGAGCAAACCACGCGACGAAGAAAGCTGGGACGAAGACGACGAAGAGTCCGAGGAAGCAGACGAAGACGGAGACTTCTAAGTGGAACTGCGGGAGAAAATCATTGAGCGAATCAAGGTGACTTCCTCTGGGTGTTGGGAGTGGCAGGGCGCTACGAACAATaa                                                                                                                                                        <  1:167262/296‑1 (MQ=255)`  
                                                                                                                                                    `actcttccggaGGGGCGGGCGTTAAGCTGCAACTGGAATCCGTGATGCTGGTCGAACTGGCTACCTTTGGTGGCGGTGAAGACGATTGGGCTGACGAAGTTGAAGAGAACGGCTATGTTGCCTCTGGTTCTGCCAAAGCGAGCAAACCACGCGACGAAGAAAGCTGGGACGAAGACGACGAAGAGTCCGAGGAAGCAGACGAAGACGGAGACTTCTAAGTGGAACTGCGGGAGAAAATCCTTGAGCGAATCAAGGTGACTTCCTCTGGGTGTTGGGAGTGGCAGGGCGCTACGAACAATaa                                                                                                                                                        <  1:153890/291‑1 (MQ=255)`  
                                                                                                                                                       `ctgctgTAGGTGCGAGCGTTAAGCTGCAACTGGAATCCGTGATGCTGGTCGAACTTGCTACCGTTGGTGGCGGTGAAGACGATTGGGCTGACGAAGTTGAAGAGAACGGCTATGTTGCCTCTGGTTCTGCCAAAGCGAGCAAACCACGCGAAGAAGAAAGCTGGGAGGAAGACGACGAAGAGTCCGAGGAAGCAGACGAAGACGGAGACTTCTAAGTGGAACTGCGGGAGAAAATCCTTGAGCGAATCAAGGTGACTTCCTCTGGGTGTTGGGAGTGGCAGGGCGCTACGAACAATAAAgg                                                                                                                                                     <  1:303787/301‑1 (MQ=255)`  
                                                                                                                                                       `ctgctgTAGGTGCGAGCGTTAAGCTGCAACTGGAATCCGTGATGCTGGTCGAACTGGCTACCTTTGGTGGCGGTGAAGACGATTGGGCTGACGAAGTTGAAGAGAACGGCTATGTTGCCTCTGGTTCTGCCAACGCGAGCAAACCACGCGACGAAGACAGCTGGGACGAAGACGACGAAGAGTCCGCGGAAGCAGACGAAGACTGAGACTTCTAAGTGGAACTGCGGGAGAAAATCCTTGAGCGAATCAAGGTGACTTCCTCTGGTTGTTGGGCGTGGCAGGGCGCTACGAACAATCAAgg                                                                                                                                                     >  1:35754/1‑301 (MQ=255)`  
                                                                                                                                                        `tgctgTAGGTGCGAGCGTTAAGCTGCAACTGGAATCCGTGATGCTGGTCGAACTGGCTACCTTTGGTGGCGGTGAAGACGATTGGGCTGACGAAGTTGAAGAGAACGGCTATGTTGCCTCTGGTTCTGCCAAAGCGAGCAAACCACGCGACGAAGAAAGCTGGGACGAAGACGACGAAGAGTCCGAGGAAGCAGACGAAGACGGAGACTTCTAAGTGGAACTGCGGGAGGAAATCCTTGAGCGAATCAAGGTGACTTCCTCTGGGTGTTGGGAGTGGCAGGGCGCTACGAACAATAAAggg                                                                                                                                                    >  1:491291/1‑301 (MQ=255)`  
                                                                                                                                                        `tgctgTAGGTGCGAGCGTTAAGCTGCAACTGGAATCCGTGATGCTGGTCGAACTGGCTACCTTTGGTGGCGGTGAAGACGATTGGGCTGACGAAGTTGAAGAGAACGGCTATGTTGCCTCTGGTTCTGCCAAAGCGAGCAAACCACGCGACGAAGAAAGCTGGGACGAAGACGACGAAGAGTCCGAGGAAGCAGACGAAGACGGAGACTTCTAAGTGGAACTGAGGGAGAAAATCCTTGAGCGAATCAAGGTGACTTCCTCTGGGTGTTGGGAGTGGCAGGGCGCTACGAACAATAAAggg                                                                                                                                                    <  1:388988/301‑1 (MQ=255)`  
                                                                                                                                                         `gctgTAGGTGCGAGCGTTAAGCTGCAACTGGAATCCGTGATGCTGGTCGAACTGGCTACCTTTGGTGGCGGTGAAGACGATTGGGCTGACGAAGTTGAAGAGAACGGCTATGTTGCCTCTGGTTCTGCCAAAGCGAGCAAACCACGCGACGAAGAAAGCTGGGACGAAGACGACGAAGAGTCCGAGGAAGCAGACGAAGACGGAGACTTCTAAGTGGAACTGCGGGAGAAAATCCTTGAGCGAATCAAGGTGACTTCCTCTGGGTGTTGGGAGTGGCAGGGCGCTACGACAAATCAAGGGt                                                                                                                                                   >  1:292448/1‑301 (MQ=255)`  
                                                                                                                                                          `ctgTAGGTGCGAGCGTTAAGCTGCAACTGGAATCCGTGATGCTGGTTGAACTGGCTACCTTTGGTGGCGGTGAAGACGATTGGGCTGACGACGTTGAAGAGAACGCCTATGATGCCTCTGGTTCTGCCAAATCGAGCAAACCACGCCACGAAGAAAGCTGGGACGAAGCCGACGAAGAGTCCGAGGAAGCAGACGAAGACGGAGACTTCTAAGTGGAACTGCGGGAGAAAATCCTTGAGCGAATCAAGGTGACTTCCTCTGGGTGTTGGGAGTGGCAGGGCGTCACGAACAATAATGGGTa                                                                                                                                                  >  1:384676/1‑301 (MQ=255)`  
                                                                                                                                                          `ctgTAGGTGCGAGCGTTAAGCTGCAACTGGAATCCGTGATGCTGGTCGAACTGGCTACCTTTGGTGGCGGTGAAGACGATTGGGCTGACGAAGTTGAAGAGAACGGCTATGTTGCCTCTGGTTCTGCCAAAGCGAGCAAACCACGCGACGAAGAAAGCTGGGACGAAGACGACGAAGAGTCCGAGGAAGCAGACGAAGACGGAGACTTCTAAGTGGAACTGCGGGAGAAAATCCTTGAGCGAATCAAGGTGACTTCCTCTGGGTGTTGGGAGTGGCAGGGCGCTACGAACAATAAAgggct                                                                                                                                                  >  1:205622/1‑299 (MQ=255)`  
                                                                                                                                                          `ctgTAGGTGCGAGCGTTAAGCTGCAACTGGAATCCGTGATGCTGGTCGAACTGGCTACCTTTGGTGGCGGTGAAGACGATTGGGCTGACGAAGTTGAAGAGAACGGCTATGTTGCCTCTGGTTCTGCCAAAGCGAGCAAACCACGCGACGAAGAAAGCTGGGACGAAGACGACGAAGAGTCCGAGGAAGCAGACGAAGACGGAGACTTCTAAGTGGAACTGCGGGAGAAAATCCTTGAGCGAATCAAGGTGACTTCCTCTGGGTGTTGGGAGTGGCAGGGCGCTACGAACAATAAAGGGTa                                                                                                                                                  >  1:307371/1‑301 (MQ=255)`  
                                                                                                                                                          `ctgTAGGTGCGAGCGTTAAGCTGCAACTGGAATCCGTGATGCTGGTCGAACTGGCTACCTTTGGTGGCGGTGAAGACGATTGGGCTGACGAAGTTGAAGAGAACGGCTATGTTGCCTCTGGTTCTGCCAAAGCGAGCAAACCACGCGACGAAGAAAGCTGGGACGAAGACGACGAAGAGTCCGAGGAAGCAGACGAAGACGGAGACTTCTAAGTGGAACTGCGGGAGAAAATCCTTGAGCGAATCAAGGTGACTTCCTCTGGGTGTTGGGAGTGGCAGGGCGCTACGAACAATAAAGGGTa                                                                                                                                                  <  1:29689/301‑1 (MQ=255)`  
                                                                                                                                                               `ggTGCGAGCGTTAAGCTGCAACTGGAATCCGTGATGCTGGTCGAACTGGCTACCTTTGGTGGCGGTGAAGACGATTGGGCTGACGAAGTTGAAGGGAACGGCTATGTTGCCTCTGGTTCTGCCAAAGCGAGCAAACCACGCGACGAAGAAAGCTGGGACGAAGACGACGAAGAGTCCGAGGAAGCAGACGAAGACGGAGACTTCTAAGTGGAACTGCGGGAGAAAAACATTGAGCGAATCAAGGTGACTTCCTCTGGGTGTTGGGAGTGGCAGGGCGCTACGAACAATAAAGGGTACgggg                                                                                                                                             >  1:501160/1‑300 (MQ=255)`  
                                                                                                                                                                 `tGCGAGCGTTAAGCTGCAACTGGAATCCGTGATGCTGGTCGAACTGGCTACCTTTGGTGGCGGTGAAGACGATTGGGCTGACGAAGTTGAAGAGAACGGCTATGTTGCCTCTGGTTCTTCCAAAGCGAGCCAACCACGCGACGAAGAAAGCTGGGAAGAAGACGCCGAAGAGTCAGATGAAGCAGACGAAGACGGAGACTTCTAAGTGGAACTGCGGGAGAAAATCCTTGCGCGAATCAAGGTGACTTCCTCTGGGGGTTGGGAGTGGCAGGGCGCTACGAACAATAAAGGGTACgctcag                                                                                                                                           >  1:504443/1‑296 (MQ=255)`  
                                                                                                                                                                  `gCGAGCGTTAAGCTGCAACTGGAATCCGTGATGCTTGTCGAACTTGCTACCTTTGGTGGCGGTGAAGACGATTGGGCTGACGAAGTTGAAGAGAACGGCTATGTTGCCTCTGGTTCTGCCAAAGCGAGCAAACCACGCGACGAAGAAAGCTGGGACGAAGACGACGAAGAGTCCGAGGAAGCAGACGAAGACGGAGACTTCTAAGTGGAACTGCGGGAGAAAATCCTTGAGCGAATCAAGGTGACTTCCTCTGGGTGTTGGGAGTGGCAGGGCGCTACGAACAATAAAGGGTACGGGcctg                                                                                                                                          <  1:195384/301‑4 (MQ=255)`  
                                                                                                                                                                            `aGCTGCAACTGGAATCCGTGATGCTGGTCGAACTGGCTACCTTTGGTGGCGGTGAAGACGATTGGGCTGACGAAGTTGAAGAGAACGGCTATGTTGCCTCTGGTTCTGCCAAAGCGAGCAAACCACGCGACGAAGAAAGCTTGTACGAAGACGACGAAGAGTCCGAGGAAGCAGACGAAGACGGAGACTTCTAAGTGGAACTGCGGGAGAAAATCCTTGAGCGAATCAAGGTGACTTCCTCTGGGTGTTGGGAGTGGCAGGGCGCTACGAACAATAAAgggctgtctcttatacacatctc                                                                                                                                >  1:327546/1‑281 (MQ=255)`  
                                                                                                                                                                            `aGCTGCAACTGGAATCCGTGATGCTGGTCGAACTGGCTACCTTTGGTGGCGGTGAAGACGATTGGGCTGACGAAGTTGAAGAGAACGGCTATGTTGCCTCTGGTTCTGCCAAAGCGAGCAAACCACGCGACGAAGAAAGCTGGGACGAAGATGACGAAGAGTCCGAGGAAGCAGACGAAGACGGAGACTTCTAAGTGGAACTGCGGGAGAAAATCCTTGAGCGAATCAAGGTTACTTCCTCTGGGTGTTGGGAGTGGCAGGGCGCTACGAACAATAAAGGGTACGGGCAGGTGTGGTgcag                                                                                                                                >  1:31104/1‑301 (MQ=255)`  
                                                                                                                                                                             `gCTGCAACTGGAATCCGTGATGCTGGTCGAACTGGCTACCTTTGGTGGCGGTGAAGACGATTGGGCTGACGAAGTTGAAGAGAACGGCTCTGTTGCCTCTGGTTCTGCCAAAGCGAGCACACCACGCGACGAAGAAAGCTGGGACGACGACGACGAAGCGTCCGAGGAAGCAGAAGCAGACGGAGACTTCTCAGTTGAACTGCGGGAGAAAATCCTTGAGCGAATCACGGTGACTTCGTCTGGGTGTTGGGAGTGGCAGCGCGCTACGAACAATACAGGGTACGGGACGGTGTGGTgcggc                                                                                                                               >  1:403909/1‑301 (MQ=255)`  
                                                                                                                                                                             `gCTGCAACTGGAATCCGTGATGCTGGTCGAACTGGCTACCTTTGGTGGCGGTGAAGACGATTGGGCTGACGAAGTTGAAGAGAACGGCTATGTTGCCTCTGGTTCTGCCAAAGCGAGCAAACCACGCGACGAAGAAAGCTGGGACGAAGACGACGAAGAGTCCGAGGAAGCAGACGAAGACGGAGACTTCTAAGTGGAACTGCGGGAGAAAATCCTTGAGCGAATCAAGGTGACTTCCTCTGGGTGTTGGGAGTGGCAGGGCGCTACGAACAATAAAGGGTACGGGCAGGAGTGGTgcagc                                                                                                                               >  1:143366/1‑301 (MQ=255)`  
                                                                                                                                                                                           `ccGTGATGCTGGTCGAACTGGCTACCTTTGGTGGCGGTGAAGACGATTGGGCTGACGAAGTTGAAGAGAACGGCTATGTTGCCTCTGGTTCTGACAAAGCGAGCAAACCACGCGACGAAGAAAGCTGGGACGAAGACGACGAAGAGTCCGAGGAAGCAGACGAAGACGGAGACTTCTAAGTGGAACTGAGGGAGAAAATCCTTGAGCGAATAAAGGTGACTTCCTCTGGGTGTTGGGAGTGGCAGGGCGCTACGAACAATAAAGGGTACGGGCAGctgtctcttatacacatctccgagcc                                                                                                                 >  1:400585/1‑279 (MQ=255)`  
                                                                                                                                                                                           `ccGTGATGCTGGTCGAACTGGCTACCTTTGGTGGCGGTGAAGACGATTGGGATGACGAAGTTGAAGAGAACGGCTATGTTGCCTCTGGTTCTGCCAAAGCGAGCAAACCACGCGACGAAGAAAGCTGGGACGAAGACGACGAAGAGTCCGAGGAAGCAGACGAAGACGGAGACTTCTAAGTGGAACTGCGGGAGAAAATCCTTGAGCGAATCAAGGTGACTTCCTCTGGGTGTTGGGAGTGGCAGGGCGCTACGAACAATAAAGGGTACGGGCAGGTGTGGTGCAGCAATCCCGGaactgt                                                                                                                 >  1:171821/1‑297 (MQ=255)`  
                                                                                                                                                                                            `cGTGATGCTGGTCGAACTGGCTACCTTTGGTGGCGGTGAAGACGATTGGGCTGACGAAGTTGAAGAGAACGGCTATGTTGCCTCTGGTTCTGCCAAAGCGAGCAAACCACGCGACGAAGAAAGCTGGGACGAAGACGACGCAGAGTCCGAGGAAGCAGACGAAGACGGAGACTTCAAAGTGGAACTGCGGGAGAACATCCTTGAGCGAATCAAGGTGACTTCCTCTGGGTGTTGGGTGTGGCAGGGCGCGACGAACAATAAAGGGCACGGGCAGGTGTGGTGCCGCAATACCGGAAAGGtt                                                                                                                >  1:498269/1‑301 (MQ=255)`  
                                                                                                                                                                                            `cGTGATGCTGGTCGAACTGGCTACCTTTGGTGGCGGTGAAGACGATTGGGCTGACGAAGTTGAAGAGAACGGCTATGTTGCCTCTGGTTCTGCCAAAGCGAGCAAACCACGCGACGAAGAAAGCTGGGACGAAGACGACGAAGAGTCCGAGGAAGCAGACGAAGACGGAGACTTCTAAGTGGAACTGCGGGAGAAAATCCTTGAGCGAATCAAGGTGACTTCCTCTGGGTGTTGGGAGTGGCAGGGCGCTACGAACAATAAA‑GGTAAGGGGAGGGGTGGTTGAGCAATTACGGGAAggccg                                                                                                               >  1:72411/1‑298 (MQ=255)`  
                                                                                                                                                                                                  `gCTGGTCGAACTGGCTACCTTTGGTTGCGGTGAAGACGATTGGGCTGACGAAGTTGAAGAGAACGGCTATGTTGCCTCTGGTTCTGCCAAAGCGAGCAAACCACGCGACGAAGAAAGCTGGGACGAAGACGACGAAGAGTCCGAGGAAGCAGACGAAGACGGAGACTTCTAAGTGGAACTGCGGGAGAAAATCCTTGAGCGAATCAAGGTGACTTCCTCTGGGTGTTGGGAGTGGCAGGGCGCTACGAACAATAAAGGGTACGGGCAGGTGTGGTGCAGCAATACCGGAAAGGTTGTCTAc                                                                                                          <  1:480825/301‑1 (MQ=255)`  
                                                                                                                                                                                                  `gCTGGTCGAACTGGCTACCTTTGGTGGAGGTGAAGACGATTGGGCTGACGAAGTTGAAGAGAACGGCTATGTTGCCTCTGGTTCTGCCAAAGCGAGCAAACCACGCGACGAAGAAAGCTGGGACGAAGAAGACGAAGAGTCCGAGGAAGCAGACGAAGACGGAGACTTCTAAGTGGAACTGCGGGAGAAAATCCTTGAGCGAATCAAGGTGACTTCCTCTGGGTGTTGGGAGTGGCAGGGCGCTACGAACAATAAAGGGTACGGGCAGGTGTGGTGCAGCAATACCGGAAAGGTTGTCTAc                                                                                                          <  1:133967/301‑1 (MQ=255)`  
                                                                                                                                                                                                     `ggTCGAACTTGCTACCTTTGGTGGCGGTGAAGACGATTGGGCTGACGAAGTTGAAGAGAACGGCTATGTTGCCTCTGGTTCTGCCAAAGCGAGCAAACCACGCGACGAAGAACGCTGGGACGAAGACGACGAAGAGTCCGAGGAAGCAGACGAAGACGGAGACGTCTAAGTGGAACTGCGGGAGAAAATCCTTGAGCGAATCAAGGTGACTTCCTCTGGGTGTTGGGAGTGGCAGGGCGCTACGAACAATAAAGGGTACGGGCAGGTGTGGTGCAGCAATACCGGAAAGGTTGTCTACTGt                                                                                                       <  1:239648/301‑1 (MQ=255)`  
                                                                                                                                                                                                              `ggCTACCTTTGGTGGCGGTGAAGACGATTGGGCTGACGAAGTTGAAGAGAACGGCTATGTTGCCTCTGGTTCTGCCAAAGCGAGCAAACCACGCGACGAAGAAAGATGGGACGCAGACGACGAAGAGTCCGATGAAGCAGACGAAGACGGAGACTTCTAAGTGGAACTGCGGGAGAAAATCCTTGAGCGAATCAAGGTGACTTCCTCTGGGTGTTGGGAGTGGCAGGGCGCTCCTAACAATAAAGGGTACGGGCAGGTGTGGTGCAGCAATACCGGAAAGGTTGTCTACTGTCATCGCGTa                                                                                              >  1:77758/1‑301 (MQ=255)`  
                                                                                                                                                                                                                  `aCCTTTGGTGGCGGTGAAGACGATTGGGCTGACGAAGTTGAAGAGAACGGCTATGTTGCCTCTGGTTCTGCCAAAGCGAGCAAACCACGCGACGAAGAAAGCTGGGACGAAGACGACGAAGAGTCCGAGGAAGCAGACGAAGACGGAGACTTCTAAGTGGAACTGCGGGAGAAAATCCTTGAGCGAATCAAGGTGACTTCCTCTGGGTGTTGGGAGTGGCAGGGCGCTACGAACAATAAAGGGTACGGGCAGGTGTGGTGCAGCAATACCGGAAAGGTTGTCTACTGTCATCGCGTAATGt                                                                                          >  1:494935/1‑301 (MQ=255)`  
                                                                                                                                                                                                                              `ggTGAAGACGATTGGGCTGACGAAGTTGAAGAGAACGGCTATGTTGCCTCTGGTTCTGCCAAAGCGAGCAAACCACGCGACGAAGAAAGCTGGGACGAAGACGACGAAGAGTCCGAGGAAGCAGACGAAGACGGTGACTTCTAAGTGGAACTGCGGGAGAAAATCCTTGAGCGAATCAAGGTGACTTCCTCTGGGTGTTGGGAGTGGCAGGGCGCTACGCACAATAAAGGGTACGGGCAGGTGTGGTGCAGCAATACCGGAAAGGTTGTCTACTGTCATCGCGTAATGTCTAATGATCCGa                                                                              >  1:257758/1‑301 (MQ=255)`  
                                                                                                                                                                                                                               `gTGAAGACGATTGGGCTGACGAAGTTGAAGAGAACGGCTATGTTGCCTCTGGTTCTGCCAAAGCGAGCAAACCACGCGACGAAGAAAGCTTGGACGAAGACGACGAAGAGTTCGAGGAAGCAGACGAAGACGGAGACTTCTAAGTGTAACTGCGGGAGAAAATCCTTGAGCGAATCAAGGTGACTTCCTCTGGGTGTTGGGAGTGGCAGGGCGCTACGAACAATAAAGGGTACGGGCAGGTGTGGTGCAGCAATACCGGAAAGGTTGTCTACTGTCATCGCGTAATGTCTAATGCTCCGaa                                                                             >  1:184319/1‑301 (MQ=255)`  
                                                                                                                                                                                                                                      `cGATTGGGCTGACGAAGTTGAAGAGAACGGCTATGTTGCCTCTGGTTCTGCCAAAGCGAGCAAACCACGCGACGAAGAAATCTGGGACGAAGACGACGAAGAGTCCGAGGAAGCAGACGAAGACGGAGACTTCTAAGTGGAACTGCGGGAGAAAATCCTTGAGCGAATCAAGGTGACTTCCTCTGGGTGTTTGGAGTGGCAGGGCGCTACGAACAATAAAGGGTACGGGCAGGTGTGGTGCAGCAATACCGGAAAGGTTGTCTACTGTCATCGCGTAATGTCTAATGCTCCTAAAGGTTCt                                                                      >  1:41246/1‑301 (MQ=255)`  
                                                                                                                                                                                                                                        `acgtgtataagagacaggAAGAGAACGGCTATGTTGCCTCTGGTTCTGGCAAAGCGAGCAGACCACGCGACGAAGAAAGCTGGGACGAAGACGACGAAGAGTCCGAGGAAGCAGACGAAGACGGAGACTTCTAAGTGGAACTGCGGGAGAAAATCCTTGAGCGAATCAAGGTGACTTCCTCTGGGTGTTGGGAGTGGCAGGGCGCTACGAACAATAAAGGGTACGGGCAGGTGTGGTGCAGCAATACCGGAAAGGTTTTCTACTGTCATCGCGTAATGTCTAATGCTCCGAAAGGTTCTAc                                                                    <  1:394565/284‑1 (MQ=255)`  
                                                                                                                                                                                                                                        `aTTGGGATGACGAAGTTGAAGAGAAAGGCTATGTTGCCTCTGGTTCTGCCAAAGCGAGCAAACCACGCGACGAAGAAAGCTGGGACGAAGACGACGAAGAGTCCGAGGAAGCAGACGAAGACGGAGACTTCTAAGTGGAACTGCGGGAGAAAATCCTTGAGCGAATCAAGGTGACTTCCTCTGGGTGTTGGGAGTGGCAGGGCGCTACGAACAATAAAGGGTACGGGCAGGTGTGGTGCAGCAATACCGGAAAGGTTGTCTACTGTCATCGCGTAATGTCTAATGCTCCGAAAGGTTCTAc                                                                    <  1:234338/301‑1 (MQ=255)`  
                                                                                                                                                                                                                                           `gggCTGACGAAGTTGAAGAGAACGGCTATTTTGCCTCTGGTTCTGCCAAAGCGAGCAAACCACGCGACGAAGAAAGCTGGGACGAAGACGACGAAGAGTCCGAGGAAGCAGACGAAGACGGAGACTTCTAAGTGGAACTGCGGGAGAAAATCCTTGAGCGAATCAAGGTGACTTCCTCTGGGTGTTGGGAGTGGCAGGGCGCTACGAACAAGAAAGGGTACGGGCAGGTGTGGTGCAGCAATACCGGAAAGGTTGTCTACTGTCATCGCGTAATGTCTAATGCTCCGAAAGGGTCTAcctt                                                                 >  1:34304/1‑299 (MQ=255)`  
                                                                                                                                                                                                                                                `gACGAAGTTGAAGAGAACGGCTATGTTGCCTCTGGTTCTGCCAAAGCGAGCAAACCACGCGACGAAGAAAGCTGGTACGAAGACGACGACGAGTCCGAGGAAGCAGACGAAGACGGAGACTTCTAAGTTCAACTGCGGGAGACCATCCTTGAGCGAGTCAAGGTGACTTCCTCTGGGGGCTGGGAGTGGCCGGGCGCTACGAACATTAAAGCGTACGTGCAGGTGTGGTGCAGCAATACCGGCAAGGTTGTCTTCTGTCATCGCGTCATGTCTATTGCTCCGAAAGTTTCTACCGTCCttc                                                            >  1:434729/1‑299 (MQ=255)`  
                                                                                                                                                                                                                                                    `aaGTTGAAGAGAACGGCTATGTTGCCTCTGGTTCTGCCAAAGCGAGCAAACCACGCGACGAAGAAAGCTGGGACGAAGACGACGAAGAGTCCGAGGAAGCAGACGAAGACGGAGACTTCTAAGTGGAACTGCGGGAGAAAATCCTTGAGCGAATCAAGGTGACTTCCTCTGGGTGTTGGGAGTGGCAGGGCGCTACGAACAATAAAGGGTACGGGCAGGTGTGGTGCAGCAATACCGGAAAGGTTGTCTACTGTCATCGCGTAATGTCTAATGCTCCGAAAGGTTCTACCGTCCTGCACTc                                                        >  1:129537/1‑301 (MQ=255)`  
                                                                                                                                                                                                                                                         `gAAGAGAACGGCTATGTTGCCTCTGGTTCTGCCAAAGCGAGCAAACCACGCGACGAAGAAAGCTGGGACGAAGACGACGAAGAGTCCGAGGAAGCAGACGAAGACGGAGACTTCTAAGTGGAACTGCGGGAGAAAATCCTTGAGCGAATCAAGGTGACTTCCTCTGGGTGTTGGGAGTGGCAGGGCGCTACGAACAATAAAGGGTACGGGCAGGTGTGGTGCAGCAATACCGGAAAGGTTGTCTACTGTCATCGCGTAATGTCTAATGCGCCGAAAGGTTCTACCGTCCTGCAcctgtctc                                                   >  1:225152/1‑294 (MQ=255)`  
                                                                                                                                                                                                                                                            `gagaACGGCGAGGTTGCCGCTGGTTCTGCCAAAGCGAGCAAACCACGCGACGAAGAAAGCTGGGACGAAGACGACGAAGAGTCCGAGGAAGCAGACGAAGACGGAGACTTCTAAGTGGAACTGCGGGAGAAAATCCTTGAGCGACTCAAGGTGACTTCCTCTGGGTGTTGGGAGTGGCAGGGCGCTACGAACAATAAAGGGTACGGGCAGGTGTGGTGCAGCAATACCGGAAAGGTTGTCTACTGTCATCGCGTAATGTCTAATGCTCCGAAAGGTTCTACCGTCCTGCACTCCTGTGATa                                                <  1:476179/301‑1 (MQ=255)`  
                                                                                                                                                                                                                                                            `aacagaggatATGTGGCGTCTGGTTCTGCCAAAGCGAGCAAACCACGCGACGAAGAAAGCTGGGACGAAGACGAAGAAGAGTCCGAGGAAGCAGACGAAGACGGAGACTTCTAAGTGGAACTGCGGGAGAAAATCCTTGAGCGAATCAAGGTGACTTCCTCTGGGTGTTGGGAGTGGCAGGGAGCTACGAACAATAAAGGGTACGGGCAGGTGTGGTGCAGCCATACCGGCAAGGTTGTCTACTGTCATCGCGTAATGTCTAATGCTCCGAAAGGTTCTACCGTCCTGCACTCCTGTGATa                                                <  1:123750/292‑1 (MQ=255)`  
                                                                                                                                                                                                                                                                 `cGGCTATGTTGCCTCTGGTTCTGCCAAAGCGAGCAAACCACGCGACGAAGAAAGCTGGGACGAAGACTACGAAGAGTCCGAGGAAGCAGACGAAGACGGAGACTTCTAAGTGGAACTGCGGGAGAAAATCCTTGAGCAAATCAAGGTGACTTCCTCTGGGTGTTGGGAGTGGCAGGGCGCTACGAACAATAAAGGGTACGGGCAGGTGTGGTGCAGCAATACCGGAAAGGTTGTCTACTGTCATCGCGTAATGTCTAATGCTCCGAAAGGTTCTACCGTCCTGCACTCCTGTGATactgtc                                           >  1:230243/1‑296 (MQ=255)`  
                                                                                                                                                                                                                                                                 `cGGCTATGTTGCCTCTGGTTCTGCCAAAGCGAGCAAACCACGCGACGAAGAAAGCTGGGACGAAGACGACGAAGAGTCCGAGGAAGCAGACGAAGACGGAGACTTCTAAGTGGAACTGCGGGAGAAAATCCTTGAGCGAATCAAGGTGACTTCCTCTGGGTGTTGGGAGTGGCAGGGCGCTACGAACAATAAAGGGTACGGGCAGGTGTGGTGCAGCAATACCGGAAAGGTTGTCTACTGTCATCGCGTAATGTCTAATGCTCCGAAAGGTTCTACCGTCCTGCACTCCTGTGATAATCCa                                           >  1:9568/1‑301 (MQ=255)`  
                                                                                                                                                                                                                                                                   `gCTATGTTGCCTCTGGTTCTGCCAAAGCGAGCAAACCACGCGACGAAGAAAGCTGGGACGAAGACGACGAAGAGTCCGAGGAAGCAGACGAAGACGGAGACTTCTAAGTGGAACTGCGGGAGAAAATCCTTGAGCGAATCAAGGTGACTTCCTCTGGGTGTTGGGAGTGGCAGGGCGCTACGAACAATAAAGGGTACGGGCAGGTGTGGTGCAGCAATACCGGAAAGGTTGTCTACTGTCATCGCGTAATGTCTAATGCTCCGAAAGGTTCTACCGTCCTGCACTCCTGTGCTAATCCAtt                                         >  1:459316/1‑301 (MQ=255)`  
                                                                                                                                                                                                                                                                   `gCTATGTTGCCTCTGGTTCTGCCAAAGCGAGCAAACCACGCGACGAAGAAAGCTGGGACAAAGACGACGAAGAGTCCGAGGAAGCAGACGAAGACGGAGACTTCTAAGTGGAACTGCGGGAGAAAATCCTTGAGCGAATCAAGGTGACTTCCTCTGGGTGTTGGGAGTGGCAGGGCGCTACGAACAATAAAGGGTACGGGCAGGTGTGGTGCAGCAATACCGGAAAGGTTGTCTACTGTCATCGCGTAATGTCTAATGCTCCGAAAGGTTCTACCGTACTGCACTCCTGTGATAATCCAtt                                         >  1:13917/1‑301 (MQ=255)`  
                                                                                                                                                                                                                                                                      `aTGTTGCCTCTGGTTCTGCCAAAGCGAGCAAACCACGCGACTAAGAAAGCTGGGACGAAGACGACGAAGAGTCAGAGGAAGCAGACGAAGACGGAGACTTCTAAGTGGAACTGCGGGAGAAAATCCTTGAGCGAATCAAGGTGACTTCCTCTGTGTGTTGTGAGTGGCAGGGCGCTACGACCAATAAAGGGTACGGGCAGGTGAGGTGCAGCAATACCGGAAAGGTTGTCTACTGTCATCGCGTACTGTCTAATGCTCCGCAAGGTTCTACCGTCCTGCACTCCTGTGCTAATCCAttctt                                      >  1:362826/1‑298 (MQ=255)`  
                                                                                                                                                                                                                                                                      `aTGTTGCCTCTGGTTCTGCCAAAGCGAGCAAACCACGCGACGAAGAAAGCTGGGACGAAGACGACGAAGAGTCCGAGGAAGCAGACGAAGACGGAGACTTCTAAGTGGAACTGCGGGAGAAAATCCTTGAGCGAATCAAGGTGACTTCCTCTGGGTGTTGGGAGTGGCAGGGCGCTACGAACAATAAAGGGTACGGGCAGGTGTGGTGCAGCAATACCGGAAAGGTTGTCTACTGTCATCGCGTAATGTCTAATGCTCCGAAAGGTTCTACCGTCCTGAACTCCTGTGATAATCCATTAtt                                      >  1:28693/1‑300 (MQ=255)`  
                                                                                                                                                                                                                                                                      `aTGTTGCCTCTGGTTCTGCCAAAGCGAGCAAACCACGCGACGAAGAAAGCTGGGACGAAGACGACGAAGAGTCCGAGGAAGCAGACGAAGACGGAGACTTCTAAGTATAACTGCGGGAGAAAATCCTTGAGCGAATCAAGGTGACTTCCTCTGGGTGTTGGGAGTGGCAGGGCGCTACGAACAATAAAGGGTACGGGCAGGTGTGGTGCAGCAATACCGGAAAGGTTGTCTACTGTCATCGCGTAATGTCTAATGCTCCGAAAGGGTCTCCCGTCCTGCACTCCTGTGCTCATCCCTTAtg                                      >  1:485110/1‑301 (MQ=255)`  
                                                                                                                                                                                                                                                                       `tgtggactctGGTTCTGCCACAGCGAGCAAACAACGCGACGAAGAACGCTGGGACGAAGACGACGAAGAGTCCGAGGAAGCAGACGAAGTCGGAGACTTCGAAGTGGAACTGCGTGAGAAAATCCTTGAGCGAATCAAGGTGACTTCCTCTGGGTGTTGGGAGTGGCAGGGCGCTACGAACAATAAAGGGTACGGGCAGGTGTGGTGCAGCAATACCGGAAAGGTTGTCTACTGTCATCGCGTAATGTCTAATGCTCCGAAAGGTTCTACCGTCCTGCACTCCTGTGATAATCCATTAtgt                                     <  1:301214/295‑1 (MQ=255)`  
                                                                                                                                                                                                                                                                         `ttGCCACTGGTTCTGCCAAAGCGAGCAAAACAAGCAACGAAGAAAGCTGGGACGAAGACGACGAAGAGTCCGAGGAAGCAGACGAAGACGGAGACTTCTAAGTGGAACTGCGGGAGAAAATCCTTGAGCGAATCAAGGTGACTTCCTCTGGGTGTTGGGAGTGGCAGGGCGCTACGAACAATAAAGGGTACGGGCAGGTGTGGTGCAGCAATACCGGAAAGGTTGTCTACTGTCATCGCGTAATGTCTAATGCTCCGAAAGGTTCTACCGTCCTGCACTCCTGTGATAATCCATTAtgttg                                   <  1:376918/301‑1 (MQ=255)`  
                                                                                                                                                                                                                                                                             `ctctGGTTCTGCCAAATCGAGCAAACCACGCGACGAAGAAAGCTGGGACGAAGACGACGAAGAGTCCGAGGAAGCAGACGAAGACGGAGACTTCTAAGTGGAACTGCGGGAGAAAATCCTTGAGCGAATCAAGGTGACTTCCTCTGGGTGTTGGGAGTGGCAGGGCGCTACGAACAATAAAGGGTACGGGCAGGTGTGGTGCAGCAATACCGGAAAGGTTGTATACTGTCATCGCGTAATGTCTAATGCTCCGAAAGGTTCTACCGTCCTGCACTCCTGTGactgtctcttatacacatct                               >  1:508401/1‑282 (MQ=255)`  
                                                                                                                                                                                                                                                                                       `gCCAAAGCGAGCAAACCACGCGACGAAGAAAGCTGGGACGAAGACGACGAAGAGTCCGAGGAAGCAGACGAAGACGGAGACTTCTAAGTGGAACTGCGGGAGAAAATCCTTGAGCGAATTAAGGTGACTTCCTCTGGGTGTTGGGAGTGGCAGGGCGATACGAACAATAAAGGGTACGGGCAGGTGTGGTGCAGCAATACCGGAAAGGTTGTCTACTGTCATCGCGTAATGTCTAATGCTCCGAAAGGTTCTACCGTCCTGCACTCCTGTGATAATCCATTATGTTGTAACCCTGAACAcc                     <  1:407568/301‑1 (MQ=255)`  
                                                                                                                                                                                                                                                                                       `gCCAAAGCGAGCAAACCACGCGACGAAGAAAGCTGGGACGAAGACGACGAAGAGTCCGAGGAAGCAGACGAAGACGGAGACTTCTAAGTGGAACTGCGGGAGAAAATCCTTGAGCGAATCAAGGTGACTTCCTCTGGGTGTTGGGAGTGGCAGGGCGCTACGAACAATAAAGGGTACGGGCAGGTGTGGTGCAGCAATACCGGAAAGGTTGTCTACTGTCATCGCGTAATGTCTAATGCTCCGAAAGGTTCTACCGTCCTGCACTCCTGTGATAATCCATTATGTTGTAACCCTGAACAcc                     >  1:25348/1‑301 (MQ=255)`  
                                                                                                                                                                                                                                                                                       `aacagaGCGAGCAAACCACGCGACGAAGAAAGCTGGGACGAAGACGACGAAGAGTCCGAAGAAGCAGACGAAGACGGAGACTTCTAAGAGGAACTTCGGGAGAAAATCCTTGAGCGAATCAAGGTGACTTCCTCTGGGTGTTGGGAGTGGCAGGGCGCTACGAACAATAAAGGGTACGGGCAGGTGTGGTGCAGCAATACCGTAAAGGTTGTCTACTGTCATCGCGTAATGTCTAATGCTCCGAAAGGTTCTACCGTCCTGCACTCCTGTGATAATCCATTATGTTGTAACCCTGAACAcc                     <  1:17955/296‑1 (MQ=255)`  
                                                                                                                                                                                                                                                                                            `aGCGAGCAAACCACGCGACGAAGAAAGCTGGGACGAAGACGACGAAGAGTCCGAGGAAGCAGACGAAGACGGTGACTTCTAAGTGGAACTGCGGGAGAAAATCCTTGAGCGAATCAAGGTGACTTCCTCTGGGTGTTGGGAGTGGCAGGGCGCTACGAACAATAAAGGGTACGGGCAGGTGTGGTGCAGCAATACCGGAAAGGTTGTCTACTGTCATCGCGTAATGTCTAATGCTCCGAAAGGTTCTACCGTCCTGCACTCCTGTGATAATCCATTATGTTGTAACCCTGAACACCTATcc                <  1:372412/301‑1 (MQ=255)`  
                                                                                                                                                                                                                                                                                            `aGCGAGCAAACCACGCGACGAAGAAAGCTGGGACGAAGACGACGAAGAGTCCGAGGAAGCAGACGAAGACGGAGACTTCTAAGTGGAACTGCGGGAGAAAATCCTTGAGCGAATCAAGGTGACTTCCTCTGGGTGTTGGGAGTGGCAGGGCGCTACGAACAATAAAGGGTACGGGCAGGTGTGGTGCAGCAATACCGGAAAGGTTGACTCCTGTCATCACGTAATGTCTAATGCTCCGAAAGGTTCTACCGTCCTGCAATACTGTGATAATCCCTTATGTTGTCACCCTGAACACCTCTcc                >  1:110233/1‑301 (MQ=255)`  
                                                                                                                                                                                                                                                                                                      `ccACGCGACGAAGAAAGCTGGGACGAAGACGACGAAGCGGCCGAGGAAGCAGACGAAGACGGAGACTTCTAAGTGGAACTGCGGGAGAAAATCCTTGAGCGAATCAAGGTGACTTCCTCTGGGTGTTGGGAGTGGCAGGGCGCTACGAACAATAAAGGGTACGGGCAGGTGTGGTGCAGCAATACCGGAAAGGTTGTCTACTGTCATCGCGTAATGTCTAATGCTCCGAAAGGTTCTACCGTCCTGCACTCCTGTGATAATCCATTATGTTGTAACCCTGAACACCTATCCATAGGAACTc      <  1:162932/301‑1 (MQ=255)`  
                                                                                                                                                                                                                                                                                                      `ccACGCGACGAAGAAAGCTGGGACGAAGACGACGAAGAGTCCGAGGAAGCAGACGAAGACGGAGACTTCTAAGTGGAACTGCGGGAGAAAATCCTTGAGCGAATCAAGGTGACTTCCTCTGGGTGTTGGGAGTGGCAGGGCGCTACGAACAATAAAGGGTACGGGCAGGTGTGGTGCAGCAATACCGGAAAGGTTGTCTACTGTCATCGCGTAATGTCTAATGCTCCGAAAGGTTCTACCGTCCTGCACTCCTGTGATAATCCATTATGTTGTAACCCTGAACACCTATCCATAGGAACTc      >  1:62694/1‑301 (MQ=255)`  

                                                                                                                                                                                                                                                                                                          `|`                                                                                                                                                                                                                                                                                                              
`GCGTCTTTCCAAGACAAGAAGACCAAAGAGACCAAGCACATCAATCTGGTTGTGGTTGACTCAAAAGGTAAGAAGATGGAAGACGTTCCGATTATCGGTGGTGGCTCTAAGCTGAAAGTTAAATATTCTCTGGTTCCATACAAGTGGAACACTGCTGTAGGTGCGAGCGTTAAGCTGCAACTGGAATCCGTGATGCTGGTCGAACTGGCTACCTTTGGTGGCGGTGAAGACGATTGGGCTGACGAAGTTGAAGAGAACGGCTATGTTGCCTCTGGTTCTGCCAAAGCGAGCAAACCACGCGACGAAGAAAGCTGGGACGAAGACGACGAAGAGTCCGAGGAAGCAGACGAAGACGGAGACTTCTAAGTGGAACTGCGGGAGAAAATCCTTGAGCGAATCAAGGTGACTTCCTCTGGGTGTTGGGAGTGGCAGGGCGCTACGAACAATAAAGGGTACGGGCAGGTGTGGTGCAGCAATACCGGAAAGGTTGTCTACTGTCATCGCGTAATGTCTAATGCTCCGAAAGGTTCTACCGTCCTGCACTCCTGTGATAATCCATTATGTTGTAACCCTGAACACCTATCCATAGGAACTCCAAA  >  NC_001604.1/9491‑10089`

  

Alignment Legend

Aligned base mismatch/match (shaded by quality score): `ATCG/ATCG` `< 3 ≤` `ATCG/ATCG` `< 10 ≤` `ATCG/ATCG` `< 21 ≤` `ATCG/ATCG` `< 34 ≤` `ATCG/ATCG` `< 38 ≤` `ATCG/ATCG`

Unaligned base: `atcg`    Masked matching base: `atcg`    Alignment gap: `‑`    Deleted base: `‑`

Reads not counted as support for junction

read\_name Not counted due to insufficient overlap past the breakpoint.

read\_name Not counted due to not crossing MOB target site duplication.
